# Self-assembling process of Oxalamide compounds and their nucleation efficiency in bio-degradable Poly(hydroxyalkanoate)s

**DOI:** 10.1038/srep13280

**Published:** 2015-08-20

**Authors:** Piming Ma, Yogesh S. Deshmukh, Carolus H.R.M. Wilsens, Michael Ryan Hansen, Robert Graf, Sanjay Rastogi

**Affiliations:** 1 School of Chemical and Material Engineering, Jiangnan University, Wuxi 214122, China; 2 Bio-Based Materials, Faculty of Humanities and Sciences, Maastricht University, P.O. Box 616 6200 MD, the Netherlands; 3 Department of Chemical Engineering, Eindhoven University of Technology, Netherlands; 4 Max Plank Institute for Polymer Science, Ackermannweg 10, D-55128, Mainz, Germany; 5 Department of Materials, Loughborough University, England (UK); 6 Interdisciplinary Nanoscience Center (iNANO) and Department of Chemistry, Aarhus University, Gustav Wieds Vej 14, DK-8000 Aarhus C, Denmark

## Abstract

One of the key requirements in semi-crystalline polyesters, synthetic or bio-based, is the control on crystallization rate and crystallinity. One of the limiting factors in the commercialization of the bio-based polyesters, for example polyhydroxyalkanoates synthesized by bacteria for energy storage purposes, is the slow crystallization rate. In this study, we show that by tailoring the molecular structure of oxalamide compounds, it is possible to dissolve these compounds in molten poly(hydroxybutyrate) (PHB), having a hydroxyvalerate co-monomer content of less than 2 mol%. Upon cooling the polymer melt, the homogeneously dispersed oxalamide compound crystallizes just below the melting temperature of the polymer. The phase-separated compound reduces the nucleation barrier of the polymer, thus enhancing the crystallization rate, nucleation density and crystallinity. The findings reported in this study provide a generic route for the molecular design of oxalamide-based compounds that can be used for enhancing nucleation efficiency of semi-crystalline bio-based polyesters.

In polymer industry, additives are used to improve both physical properties and functional behavior of polymers. One of the most successful examples of such additives are their application as nucleating agents to enhance crystallization rate or to alter the optical haze^1^. The decrease in solidification time of (semi-)crystalline polymers by increasing crystallization temperatures and rates, is very much desired for economic reasons. For an example, the presence of nucleating agents in polymer processing shortens the polymer processing cycle time, controls the polymer morphology, and retains the shape of the final product. However, compared to synthetic polymers, these concepts have so far not been that successful in biopolymers such as poly(hydroxyalkanoates) (PHAs). One very well-studied example of PHAs is poly (hydroxybutyrate) (PHB)^2^. Although PHAs have been commercialized in the late 80 s, its use is still limited due to several drawbacks, including thermal degradation, brittle mechanical behavior, poor nucleation, and slow crystallization rates^3^,^4^. For this reason, nucleating agents (NA) are required to reduce the nucleation barrier and thereby stimulate the crystal growth, which will subsequently enhance the crystallinity^5^.

So far, several inorganic and organic additives such as cyanuric acid^6^, uracil^7^ and boron nitride^8^, amongst others^9^,^10^,^11^,^12^,^13^,^14^,^15^,^16^,^17^,^18^, are reported as nucleating agents for PHB and its copolymers. However, in most of these studies, the polymer and nucleating agents exhibit only effective nucleation at slow cooling rates, typically at 10 °C/min. Among these NAs, inorganic boron nitride has been commercially used in the processing of PHB and PHBV, showing high nucleation efficiency. Similarly, the organic compounds cyanuric acid and uracil have been reported to exhibit high nucleating efficiency for PHB^6^,^7^. The required slow cooling rates for the good nucleation efficiency are deterrent to the fast processing conditions demanded for industrial applications.

One of the key requirements of good nucleating agents is its ease in miscibility in polymer melt. However, most of the reported nucleating agents are not miscible with the PHB melt, which causes inhomogeneous dispersion of the additive in the PHB matrix limiting the nucleation efficiency. To improve the nucleation efficiency, a nucleating agent having good miscibility in the melt state of PHB is desired. Ideally, upon cooling, the miscible compound should phase separate (liquid-solid transition) just below the equilibrium melting temperature of the polymer and consecutively stimulate heterogeneous nucleation for crystal growth. As a result, the crystallization temperature and growth upon cooling is likely to increase. Commercial examples that follow this concept in the nucleation of PP are the sorbitol based NAs, Irgaclear^®^, NA11, amongst others^19^,^20^,^21^,^22^,^23^,^24^,^25^. In this paper, we report on the development of a series of oxalamide compounds that show good miscibility with PHB during melt-processing, which promote the crystallization upon cooling of PHB to higher temperatures. Furthermore, the design of oxalamide compounds is discussed, and the nucleation efficiency of these compounds is tailored for different polymers by changing their molecular architecture.

## Materials and Experimental methods

### Materials

**Figure 1:**
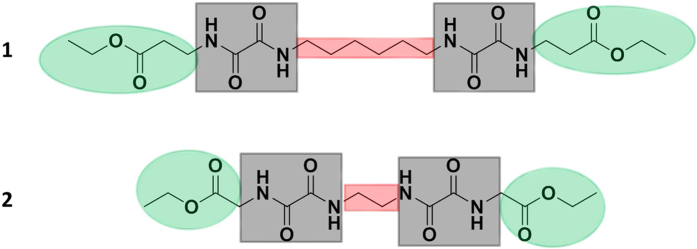
Chemical structure of the two oxalamides, diethyl 5, 6,15,16-tetraoxo-4,7,14,17-tetraazaicosanedioate (**1**) and diethyl 4,5,10,11-tetraoxo-3,6,9,12-tetraazatetradecanedioate (**2**). The molecular design concept of the nucleating agent in compound **1** and **2** is represented by color scheme. Green represents the end groups that help to solubilize the NA in the polymer matrix, grey represents oxalmide groups which promotes the self-assembling process of the compounds in the polymer melt, and red, represents the linker molecules (aliphatic spacers) which governs the melting of NA.

All chemicals used in the synthesis of the compounds were obtained from Sigma-Aldrich and were used as received. The synthesis of the oxalamide compounds *diethyl 4,5,14,15-tetraoxo-3,6,13,16-tetraazaoctadecane-1,18-dioate* (**1**) and *diethyl 4,5,10,11-tetraoxo-3,6,9,12-tetraazatetradecanedioate* (**2**) was carried out in two steps as is described in one of our previous publications^26^. Prior to the recording of DSC and solid-state NMR experiments, the compound **1** was recrystallized from super-heated water to improve the crystal perfection^27^,^28^. Bacterially synthesized PHB powder, having 2 mol% of hydroxyl valerate, was provided by Tianan Biologic Material Co. Ltd., Ningbo, China, with *M*_n_ of 156 kg/mol (PDI = 2.4, optical rotation 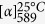 = −2.6° (2.4 g/100 ml, CHCl_3_). The oxalamide compounds used for these studies are abbreviated as compounds 1 and 2. The molecular configuration of the two compounds, 1 and 2, are presented in Fig. 1.

### Sample preparation

PHB, and the compounds **1** and **2,** were dried in a vacuum oven at 60 °C for 12 hours prior to use. After drying, the materials were melt-blended at 190 °C using a calibrated twin-screw mini-extruder at a mixing speed of 90 rpm and residence time of 5 minutes. The amount of nucleating agent in the polymer was varied between 0 wt% to 10 wt%. All experiments have been performed in duplo to ensure repeatability.

### Differential Scanning Calorimetry (DSC)

The phase transformations of the compound **1** were studied under a nitrogen rich atmosphere by DSC using a TA Q1000 instrument. Heating and cooling rates of all samples were 5 °C/min and were held for 3 min under isothermal condition at the limiting temperatures.

Water crystallized oxalamide compounds **1**: To overcome the barrier of crystallization history the oxalamide compounds were dissolved in water (under pressure) at 150 °C for 3 mins and subsequently crystallized on cooling. The crystals obtained as suspension were filtered at room temperature and dried in vacuum oven at 40 °C.

The crystallization and melting behavior of PHB, with and without the compounds **1** and **2**, was investigated by DSC under a nitrogen rich atmosphere. For isothermal crystallization experiments, to erase the thermal history, the samples were kept at 190 °C in melt state for 3 min. After giving the thermal treatment, the samples were cooled at 60 °C/min to the set isothermal crystallization temperature between 95 and 125 °C for the required crystallization time. For non-isothermal crystallization experiments, the samples were heated to 190 °C at 20 °C/min and held at 190 °C for 3 min. Subsequently, the samples were cooled to 20 °C at the maximum cooling rate of the DSC apparatus, which is approximately 60 °C/min.

### Polarized Optical Microscopy (POM)

The spherulitic morphology of PHB, with and without the compounds **1** or **2**, was studied using a Zeiss LM Axioplan microscope under crossed polarizers with a CD achorplan objective (Zoom). Following the procedure similar to DSC, the samples were heated from room temperature to 190 °C at 50 °C/min. After 3 min at 190 °C, the samples were cooled at the maximum cooling rate achievable in the DSC, approximately 60 °C/min. The morphological changes during the heating and the cooling cycles were monitored. To avoid any oxidation the experiments were performed in rich nitrogen atmosphere.

### Solid State Nuclear Magnetic Resonance (NMR)

Variable-temperature (VT) ^13^C{^1^H} magic angle spinning/cross-polarization (CP/MAS) NMR experiments were carried out on a Bruker DSX-500 spectrometer using a double resonance probe for rotors with 4.0 mm outside diameter. The experiments were performed at 10 kHz MAS using a 4 μs π/2 pulse for ^1^H. All VT ^13^C{^1^H} CP/MAS NMR spectra were recorded using a CP contact time of 3.0 ms and high-power Two Pulse Phase Modulation (TPPM) ^1^H decoupling during acquisition^29^. The temperature was controlled using a Bruker temperature control unit in the range from 30 to 210 °C. The VT ^13^C{^1^H} CP/MAS NMR spectra were recorded under isothermal conditions at intervals of 10 °C, employing a heating rate of 2 °C/min between temperatures. Reported temperatures are corrected for friction induced heating due to spinning using ^207^Pb MAS NMR of Pb(NO_3_)_2_ as a NMR thermometer^30^.

## Results and Discussion

### Self-assembling process and thermal behavior of oxalamide compounds

**Figure 2:**
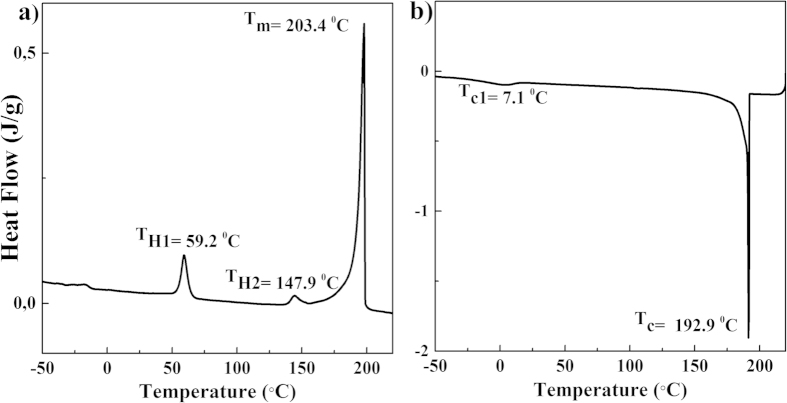
First DSC heating (**a**), and cooling (**b**) of the water-crystallized compound **1**. DSC experiments were performed at heating and cooling rates of 10 °C/min. Two endothermic peaks are observed prior to melting, which are attributed to crystal-crystal phase transformations. *N.b.* Phase transitions, during the second heating and cooling runs, were observed at similar temperatures.

**Figure 3:**
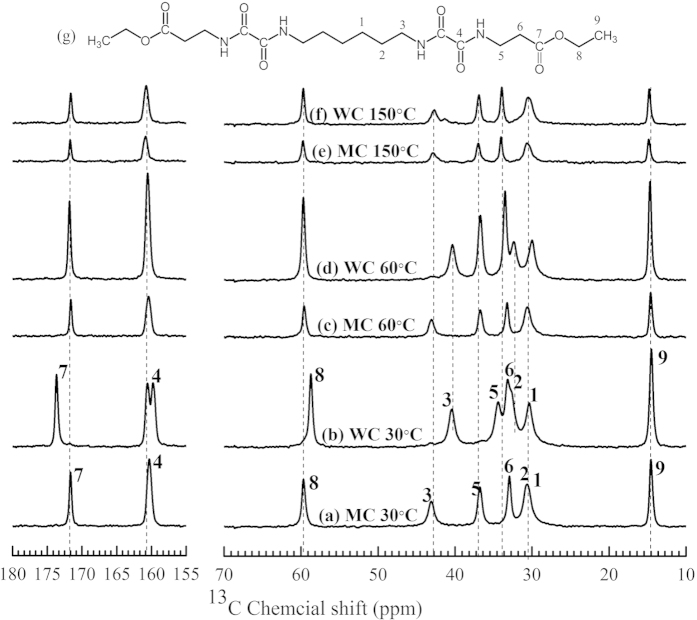
^13^C {^1^H} CP/MAS NMR spectra of compound **1** at 30 °C (**a**,**b**), 60 °C (**c**,**d**) and 150 °C (**e**,**f**) observed during the first (water-crystallized, WC) and second (melt-crystallized, MC) heating runs. The numbers at each peak positions correspond to the carbons labeled for the model compound shown on top of the figure. Dotted lines are added to visualize the changes in the ^13^C chemical shift for the samples crystallized from water or melt at 30 °C and 150 °C. The changes in ^13^C chemical shift of compound **1** are summarized in Table S2 of the Supplementary Section.

In general, the prerequisites for an efficient nucleating agent (NA) are good solubility in the polymer melt, followed by self-assembly upon cooling prior to crystallization of the polymer, where the self-assembled structures provide heterogeneous nucleation^23^,^31^. In general, secondary interactions between the separated NA molecules are required to enhance the supra-molecular aggregation process. In the oxalamide compounds, used in this study, the driving force for aggregation is the formation of hydrogen bonds. The oxalamide motif consists of two amide groups next to each other in reverse fashion^32^. The formation of two hydrogen bonds per oxalamide motif results in an increased tendency to crystallize into a β-sheet like structure^33^. The molecular structure of the oxalamide compounds (**1** and **2**, see Fig. 1) are designed in such a way that they contain both hard and soft segments. The methylene units in the center and at the end of the compounds represent the soft segments, whereas the oxalamide moieties having hydrogen bonding groups are considered to be the hard segments. It is anticipated that the melting temperature and the solubility of these compounds is dependent on the chemical architecture of the soft segments, even though the self-aggregation process of these compounds is governed by the formation of hydrogen bonding. These concepts have been further validated by following the thermal behavior and conformational changes occurring during heating of the compound **1** by DSC and solid-state NMR, respectively. Figure 2 shows the first heating (a) and cooling (b) runs of compound **1** as observed by DSC on heating at 10 °C/min, whereas Fig. 3 shows the ^13^C {^1^H} CP/MAS NMR spectra of compound **1** recorded at various temperatures during the first and the second heating runs.

On heating the compound **1**, three endothermic transitions are observed at 59.2 °C, 147.9 °C and 203.4 °C. The enthalpies of these transitions are listed in the Supplementary Section Table S1. Considering the small amount of heat involved during the endothermic transitions at 59.2 °C and 147.9 °C, these phase transitions are attributed to crystal-crystal transformations. The third transition at 203.4 °C is attributed to melting of the crystals. Upon cooling from melt (205 °C), two exothermic peaks at 192.9 °C and ~ 7.1 °C are observed. The phase transition at 192.9 °C is attributed to crystallization from melt, whereas the low temperature exothermic transition, 7.1 °C, is attributed to reorganization of the crystalline state to achieve the equilibrium state^34^. The recorded phase transitions and respective enthalpy of melt and water crystallized samples are summarized in Table S1 (see Supplementary Section).

On heating the water crystallized compound **1** to 60 °C resulted in a shift of the ^13^C resonances for the end-group (carbons 5, 7 and 8), the carbonyl resonance of the oxalamide moiety (carbon 4) and the chemical shift value of linker (carbon 1 and 2), see Fig. 3. These observations indicate that the transition at 59.3 °C corresponds to weakening of hydrogen bonding for the oxalamide motifs. The weakening is due to release in the constrained motion of the carbon next to the oxalamide group, combined with an increased mobility of the end-groups. Similarly, the transition at 146.5 °C corresponds to structural reorganization induced by an increased mobility of the flexible linker, as indicated by the shift in resonance of the carbon signals 1, 2, and 3. Simultaneously, a further enhancement of mobility is observed for the end-groups, indicated by the shift in resonance signals of the carbon labeled 6. It is anticipated that upon further heating, the enhanced mobility of the flexible components and decrease in hydrogen bonding eventually results in melting of the crystals. Although similar transitions are observed for melt crystallized sample, MC **1**, in the DSC analysis, the changes observed are less apparent from the NMR analysis. We attribute these differences in the melt and the water crystallized samples to the formation of more ordered crystal structure (with lesser defects) obtained from solvent (water). The differences in crystal structure of the water and the melt crystallized samples are discussed in the Supplementary Sections, S.2.1 and S.3.

The data presented in Figs 2 and 3 confirms that the thermal transition systematically changes the molecular conformation, and melting is initiated by increase in mobility of the flexible components. Thus by selecting length of the linking spacer, dissolution or melting temperature of an oxalamide compound can be controlled.

### Nucleation efficiency of oxalamide compounds on the crystallization behavior of PHB

**Figure 4:**
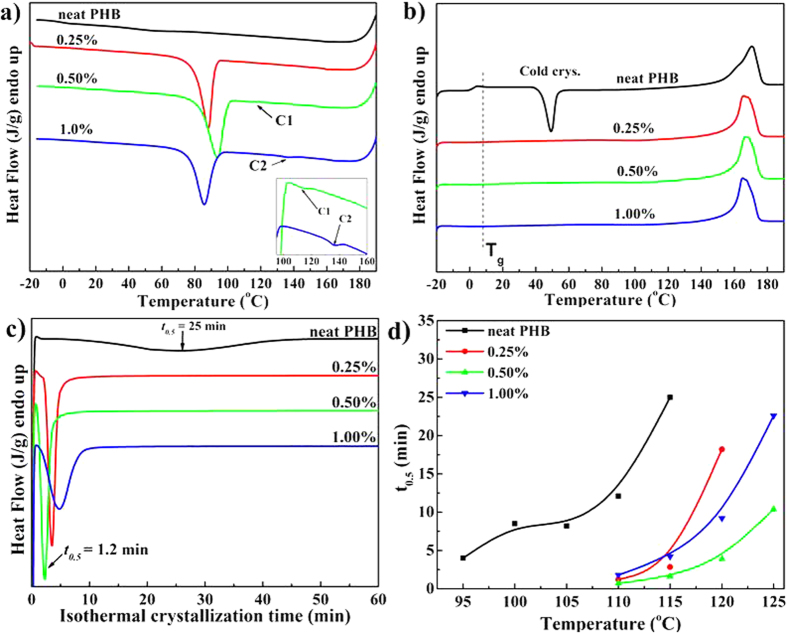
(**a**) Cooling and (**b**) subsequent heating DSC curves of neat and nucleated PHB from its melt state obtained on maximum cooling achievable in the DSC (approximately 60 °C/min). (**c**) Isothermal crystallization curves of the neat and nucleated PHB recorded at the isothermal crystallization temperature 115.0 °C for an hour, whereas (**d**) shows an overview of the crystallization half-time (t_0.5_) at different isothermal crystallization temperatures for three concentrations of the compound **2**. t_0.5_ is determined from peak of the exothermic transition time recorded during isothermal crystallization experiments.

The melting and crystallization behavior of the oxalamide compounds were demonstrated in the section above. In this section, miscibility of the oxalamides in the PHB polymer melt and crystallization kinetics of the polymer in the presence of these compounds is investigated. Recently, Ma and coworkers have demonstrated that oxalamide based compounds having aromatic end-groups are efficient nucleating agents for poly(lactic acid), during slow cooling (10 °C/min) or under isothermal conditions (135 °C)^35^. Unexpectedly, attempts to improve the nucleating efficiency of PHB with the nucleating agents developed by Ma and coworkers did not significantly enhance the nucleation and crystal growth process^36^. Considering the design principles discussed earlier, it is prerequisite to incorporate mobile end-groups that are easily dissolved in the polymer matrix. Therefore, to further promote dissolution of the oxalamide compound in the polymer matrix, the chemical structure of compound **1** is modified by changing the end-groups to resemble the repeat unit of the PHB polymer, resulting in compound **2**, see Fig. 1. Similarly, to promote phase separation of the nucleating agent at temperatures close to the crystallization of the polymer, the length of the flexible spacer connecting the oxalamide moieties has been decreased. These modifications in the molecular architecture of the compound **1** are required to meet the prerequisite of a good nucleating agent, i.e., in terms of dissolution in the polymer matrix and subsequent liquid-solid phase transition close to the equilibrium melting temperature, on cooling. The synthesized compound **2** was melt-mixed with PHB at different concentrations (0 wt% to 10 wt%) at 190 °C in a twin-screw extruder for 5 minutes. Figure 4a,4b depict heating and cooling DSC traces of the samples having 0, 0.25, 0.5, and 1 wt% of compound **2** recorded at a constant heating rate of 20 °C/min and cooling rate 60 °C/min. Figure 4c comprises DSC traces of the same samples obtained during isothermal crystallization at 115 °C. Figure 4d shows crystallization half-time (t_0.5_) of the samples with different concentrations of the compound **2**, obtained at different isothermal crystallization temperatures.

From Fig. 4a it is evident that neat PHB shows low crystallinity (*X*_c_ = ~ 2%) and slow crystallization because of low nucleation density. However, upon heating cold crystallization (*T*_cc_) is observed, Fig. 4b. In contrast, PHB mixed with compound **2** undergoes fast crystallization on cooling from melt and shows no *T*_cc_ during heating. In the presence of compound **2**, the crystallization temperature (*T*_c_) increases by 30 to 40 °C. Furthermore, the nucleated PHB show significantly larger crystallization enthalpy (Δ*H*_c_) compared to the Δ*H*_c_ for pure PHB, indicating that the total crystallinity of PHB increases in the presence of compound **2**. These results clearly show that the overall crystallization rate of PHB can be enhanced significantly in the presence of the compound **2**. Figure 4c further confirms decrease in the crystallization half time in the presence of the nucleating agent. The shift in the crystallization temperature to higher values for the same crystallization time, in the presence of nucleating agent, is further evident from Fig. 4d.

**Figure 5:**
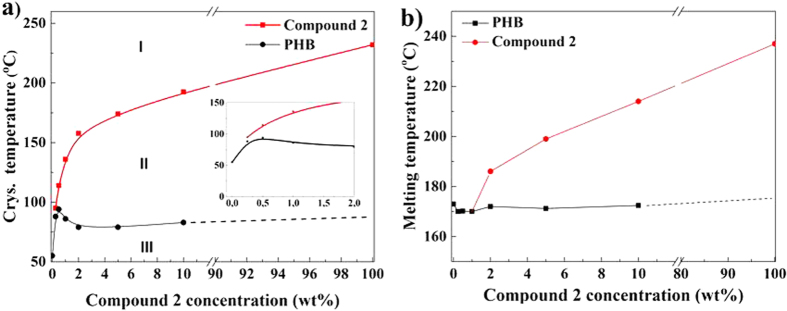
(**a**) Phase diagram of PHB with compound **2** (nucleating agent) recorded on cooling. Three different regions I, II and III are identified. Region I: homogenous phase (PHB and compound **2** are in the molten state); Region II: the molten state of PHB and solid state of compound **2**; Region III: crystallized PHB and compound **2**. The inset illustrates magnified view at low concentrations. (**b**) Phase diagram recorded on melting of the PHB/compound 2 blend.

Interestingly, in the samples having higher concentration of the nucleating agent a small exotherm (labeled C_1_ and C_2_) is observed prior to the crystallization of the polymer Fig. 4a. The transition corresponds to crystallization of the compound **2** in the polymer melt. The transition for different nucleating agent concentration is followed, and summarized in Fig. 5a. Figure 5b depicts melting temperatures of both PHB and the nucleating agent during heating.

**Figure 6:**
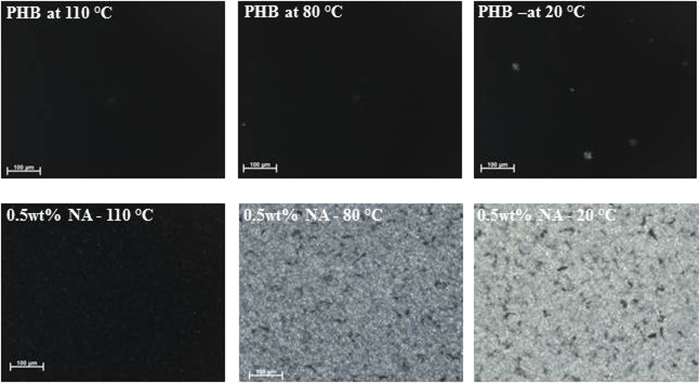
Optical micrographs of PHB viewed between cross-polars with and without compound 2; (top row) 0 wt% of compound 2 and (bottom row) with 0.5 wt% of compound 2. The samples were first melted at 190 °C for 3 min and then cooled from melt to room temperature at approximately 60 °C/min. The images were recorded at different temperatures, shown on the micrographs, during cooling.

Following the Flory-Huggins miscibility concepts, the reduction in crystallization and dissolution temperatures of the polymer as well as the nucleating agent at low concentrations of the compound, Fig. 5, conclusively demonstrates good miscibility. Such a melting temperature depression is characteristic for efficient melt-soluble nucleating agents, e.g., 1,3:2,4-bis(3,4-dimethylbenzylidene) sorbitol and Irgaclear^®^ in polypropylene^37^,^38^. From the inset picture in Fig. 5a, the crystallization temperature for PHB tends to decrease for nucleating agent concentration greater than 0.5 wt%. The decrease in the crystallization temperature, above 0.5 wt%, is associated with the self-organization of compound **2** into larger needle shaped aggregates. In this case, the formation of large aggregates decreases the surface to volume ratio required for the heterogeneous nucleation thus shifting the peak crystallization temperature to lower values. The crystallization process of pure PHB and the sample containing 0.5 wt% of compound **2** was monitored by polarization optical microscopy at a cooling rate of 60 °C/min, (see Fig. 6).

From Fig. 6 it is apparent that even at the high cooling rate, compound **2** is still capable of nucleating the PHB polymer effectively, achieving a higher crystallinity after cooling. These observations are in accordance with DSC data, Fig. 4a, where on heating the sample 0.5 wt%NA-20 °C no glass transition and cold crystallization is observed. These results strongly suggest that the synthesized oxalamide compounds can be used effectively as nucleating agent for PHB at cooling rates required for industrial processing. Further studies on the development of nucleating agents for polyhydroxyalkanoates having higher content of copolymers are in progress.

## Conclusion

In this study it is conclusively demonstrated that oxalamide based compounds can be used as nucleating agents for PHB. With the help of solid-state NMR and DSC, it is demonstrated that the thermal behavior and conformational changes can be controlled and influenced by tailoring the chemical architecture. Though the exact mechanism of nucleation for these oxalamide compounds is still under investigation, the paper addresses importance of the molecular design of the nucleating agents for their miscibility in polymer melt, phase separation from melt and self-assembling prior to crystallization of the polymer (required for the heterogeneous nucleation). These concepts, of generic nature, can be extended to other bio-based as well as synthetic polyesters and polyolefins^36^,^39^,^40^.

## Additional Information

**How to cite this article**: Ma, P. *et al.* Self-assembling process of Oxalamide compounds and their nucleation efficiency in bio-degradable Poly(hydroxyalkanoate)s. *Sci. Rep.* **5**, 13280; doi: 10.1038/srep13280 (2015).

## Supplementary Material

Supplementary Information
