# Supplementary material for: Self-assembling process of Oxalamide compounds and their nucleation efficiency in bio-degradable Poly(hydroxyalkanoate)s
Source: Sci Rep. 2015 Aug 20;5:13280. doi: 10.1038/srep13280 (PMC4642526; doi:10.1038/srep13280)
Supplement: Supplementary Information [file srep13280-s1.pdf]

Supplementary information

Self-assembling process of Oxalamide compounds and their nucleation efficiency in bio-degradable Poly(hydroxyalkanoate)s

Piming Ma[2,3,*], Yogesh. S. Deshmukh[1,3,*,#] , , Carolus H.R.M. Wilsens [3], Michael Ryan Hansen [5,7], Robert Graf [5], Sanjay Rastogi [1,3,4,6,#]

[1]BioBased Materials, Faculty of Humanities and Sciences, Maastricht University, P.O. Box 616 6200 MD, the Netherlands, [2]School of Chemical and Material Engineering, Jiangnan University, Wuxi 214122, China, [3]Department of Chemical Engineering, Eindhoven University of Technology, Netherlands, [4]Research Institute, Teijin Aramid, Arnhem, Netherlands, [5]Max Plank Institute for Polymer Science, Ackermannweg 10, D-55128, Mainz, Germany, [6]Department of Materials, Loughborough University, England (UK). [7]Interdisciplinary Nanoscience Center (iNANO) and Department of Chemistry, Aarhus University, Gustav Wieds Vej 14, DK-8000 Aarhus C, Denmark.

Section S.1 Self-assembling process and thermal behavior of oxalamide compounds.

Table S1. Summary of phase transition temperatures and enthalpy involved during the transition of Compound **1**

	NA1	
	MC	WC
T_1 (°C)	59.3	59.2
ΔH_1 (J/g)	16.3	14.4
T_2(°C)	146.5	147.9
ΔH_2(J/g)	4.8	5.1
T_m(°C)	200.2	203.4
ΔH_m(J/g)	131.2	137.9
T_c(°C)	194.3	192.9
ΔH_c(J/g)	133.8	135.8
T_{c1}(°C)	6.8	7.1
ΔH_{c1}(J/g)	24.8	26.1
T_{c2}(°C)	A	A
ΔH_{c2}(J/g)	A	A

[A] absent

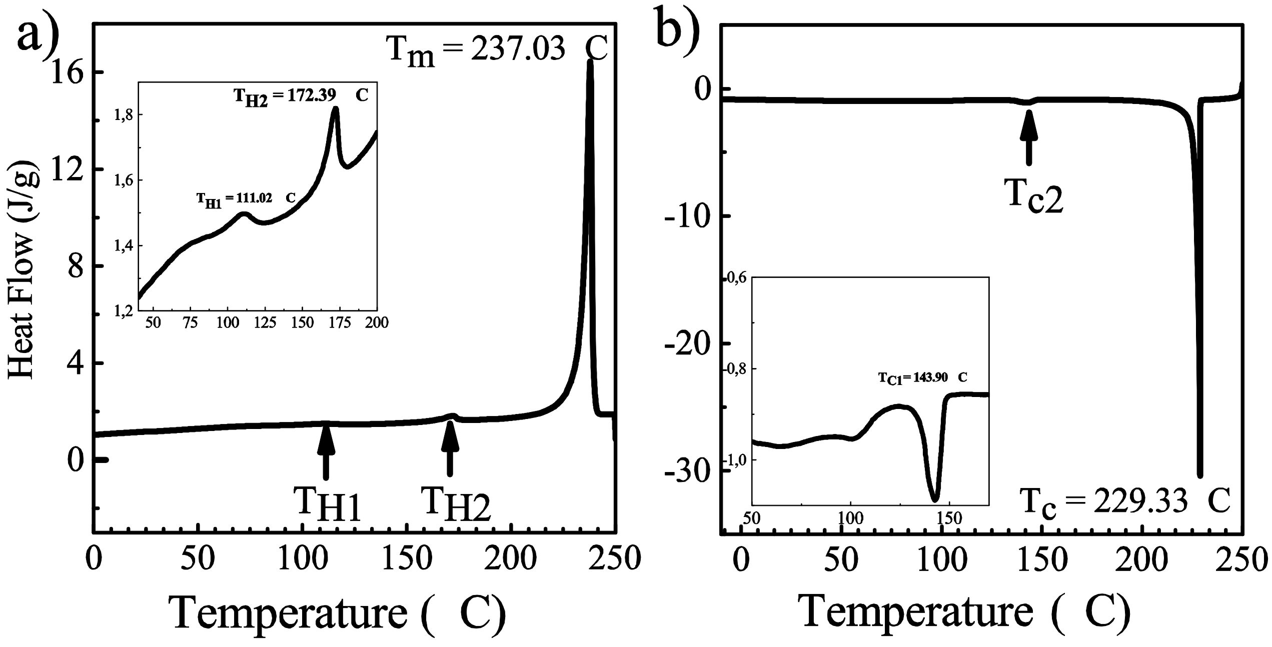

Figure S1. First DSC heating (a) and cooling (b) of the melt-crystallized compound **2**. DSC experiments were performed at heating and cooling rates of 10 °C/min. Similar to compound **1,** in compound **2, t**wo endothermic peaks are observed prior to melting, which are attributed to crystal-crystal phase transformations. The endothermic peaks are observed at higher temperature,indicates better crystal packing due to changes in molecular structure (see Figure 1) *N.b.* Phase transitions, during the second heating and cooling runs, were observed at similar temperatures.

Section S. 2. Solid-state $^{13}C\{^{1}H\}$ CP/MAS NMR spectroscopy

Table S2: *Changes in ^{13}C chemical shift values of NA1 crystallized from melt and water at elevated temperature*

	^{13}C Chemical Shift (ppm)								
	7	4	3	1	2	6	5	8	9
MC $^{T↑}$									
30°C	171.7	160.3	43.0	30.9	30.9	32.9	36.8	59.4	14.5
70°C	171.7	160.4	43.0	30.7	30.7	33.2	37.0	59.6	14.5
140°C	171.7	160.9	42.8	30.8	30.8	33.7	37.0	59.7	14.7
170°C	171.7	160.9	42.7/41.6	30.6	31.2	34.1	37.0	59.9	14.9
WC $^{T↑}$									
30°C	173.8	160.5/159.7	40.1	30.2	32.5	33.1	34.5	58.7	14.4
70°C	171.8	160.3	40.3	30.1	32.4	33.4	36.7	59.7	14.7
140°C	171.7	160.7	42.7/41.6	30.5	33.9	33.9	36.9	59.7	14.7
170°C	171.7	160.8	41.6	29.9	31.4	34.0	37.9	59.8	14.7

[a] Assignment of the individual carbon atoms can be seen in Figure 3.

S.2.1 2D $^{1}H-^{1}H$ double quantum-single-quantum (DQ-SQ) correlation spectroscopy

The 2D ^{1}H-^{1}H Double-Quantum Single-Quantum (DQ-SQ) correlation experiments were recorded on a Bruker AVANCE-III 850 spectrometer (υ_L = 850.27 MHz for ^{1}H) using a double-resonance probe for rotors with 2.5 mm outside diameter. Experiments were performed under rotor-synchronized conditions using a spinning frequency of 29762 Hz. The BaBa sequence was used for excitation and reconversion of DQ coherences [i,ii]. All 2D spectra were recorded using two rotor periods (67.2 μs) of BaBa DQ recoupling. Chemical shifts for ^{1}H and ^{13}C are reported in ppm relative to TMS using solid adamantane as an external reference [iii,iv]. From a comparison between the data in Table S2, and Figures S3 (a) and Figure S3 (b), it is apparent that considerable differences exist in the chemical environment of the oxalamide groups, where the origin of these differences is attributed to the crystallization conditions. Important to notice is that, unlike the ^{13}C peaks of the oxalamide groups and the neighboring CH_2 units, the ^{13}C peak position of the CH_3 end group unaffected by the crystallization conditions. Moreover, the ^{13}C peak of the CH_2 unit in the middle of the

molecule, number 1, remains independent of the crystallization conditions. Comparison between the melt and the water crystallized samples show a very small shift in the CH_2 peak of carbon number 2. However, dramatic differences are observed for the remainder of the carbon resonances associated with the hydrogen bonding oxalamide groups (^{13}C peak positions 3, 4, and 5) and the carbonyl groups (^{13}C peak position 7).

From these signals it is apparent that hydrogen bonding in the solid state is strongly influenced by the crystallization conditions. In the sample crystallized from superheated water a possibility of the presence of water molecules within the lattice cannot be excluded. The involvement of water molecules and their strong interaction with the hydrogen bonding groups may influence the ^{13}C chemical shifts. Such a possibility may explain the remarkable differences in the peak positioning of the ^{13}C oxalamides, crystallized from the melt or from super-heated water. To check such a possibility 2D ^{1}H–^{1}H double quantum-single-quantum (DQ-SQ) correlation spectroscopy have been performed on the water and melt crystallized samples and the results are depicted in Figure S3.

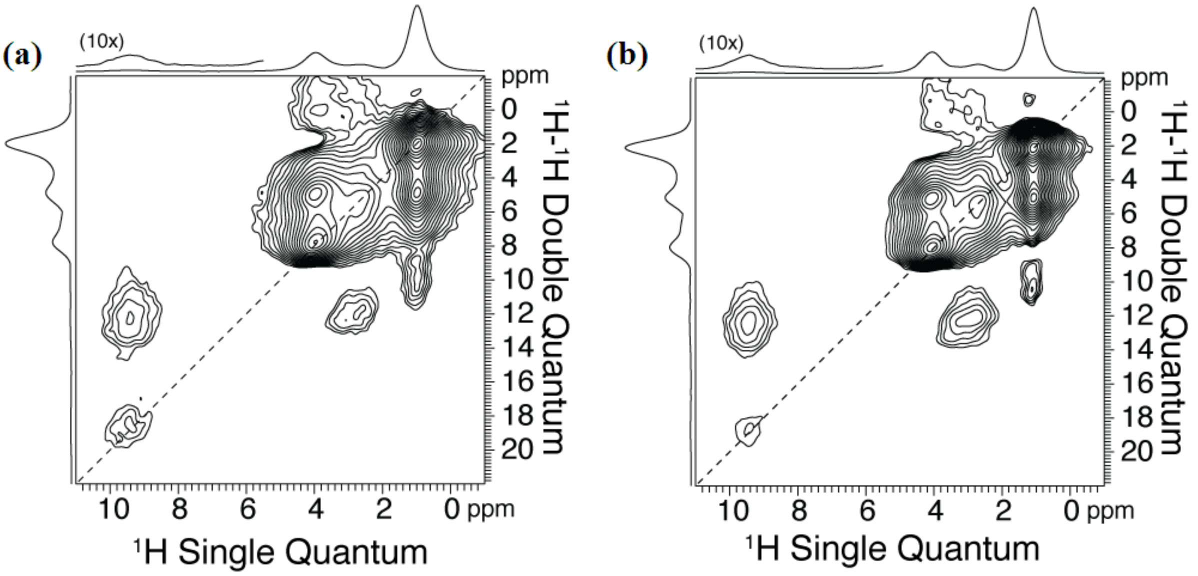

Figure S3: 2D rotor-synchronized ^{1}H-^{1}H DQ-SQ correlation spectra of (a) melt and (b) water crystallized **1** recorded at ambient conditions.

Within the available resolution limit of the 850 MHz spectrometer, no considerable differences can be observed in the ^{1}H spectra of the melt and the water crystallized samples. The absence of any water molecules, again within the experimental resolution limit, excludes the influence of water molecules on the hydrogen bonding of the oxalamide groups. Thus we attribute the differences observed in the $^{13}C\{^{1}H\}$ CP/MAS NMR spectra of the water and the melt crystallized samples, depicted in Figure 3 of the manuscript, to an ease in packing of the macromolecules facilitated by the presence of water, where the latter acts as plasticizer in the structuring process.

Section S.3. Fourier Transform Infra-Red spectroscopy (FTIR).

The NMR studies are further complemented by the FTIR spectroscopy experiments performed on compound **1**. Figures 4S (a) and Figure S4 (b) summarizes FTIR spectra recorded on the melt and water crystallized samples. Unlike the NMR spectra the FTIR spectra of the starting material looks similar. No differences in the positioning of the vibrational modes are observed. However, the water crystallized sample shows broader peaks in the region of 1000-1400 cm^{-1} compared to the melt crystallized sample. The broad peak in FTIR suggests broader distribution in the vibrational modes. With the onset of the transition T_{H1} in the vicinity of 60 °C, changes are observed in the spectral region of 1000-1400 cm^{-1}. To be more specific the vibrational modes associated with the carbonyl group outside the oxalamide moieties show a sudden peak broadening. These findings are in accordance with the changes registered by the NMR spectra in the water crystallized sample. Changes in the region around 800 cm^{-1} in both melt and water crystallized samples are also registered. These are again attributed to the conformational changes of the free carbonyl group.

Conformational changes in compound **1,** crystallized from the melt and from water, were studied by FTIR spectroscopy. The samples were placed on a zinc selenium disk and spectra were recorded on a FTS670IR spectrometer equipped with a microscope with a resolution of 4 cm^{-1} in the transmission mode. The samples were heated and cooled at rates of 5 °C/min. FTIR spectra were collected at intervals of 10 °C and obtained after averaging 100 spectra. The recording was initiated after leaving the sample for 3 min under the isothermal condition. Linkam TMS94 hotstage was used to follow the heating and cooling cycles.

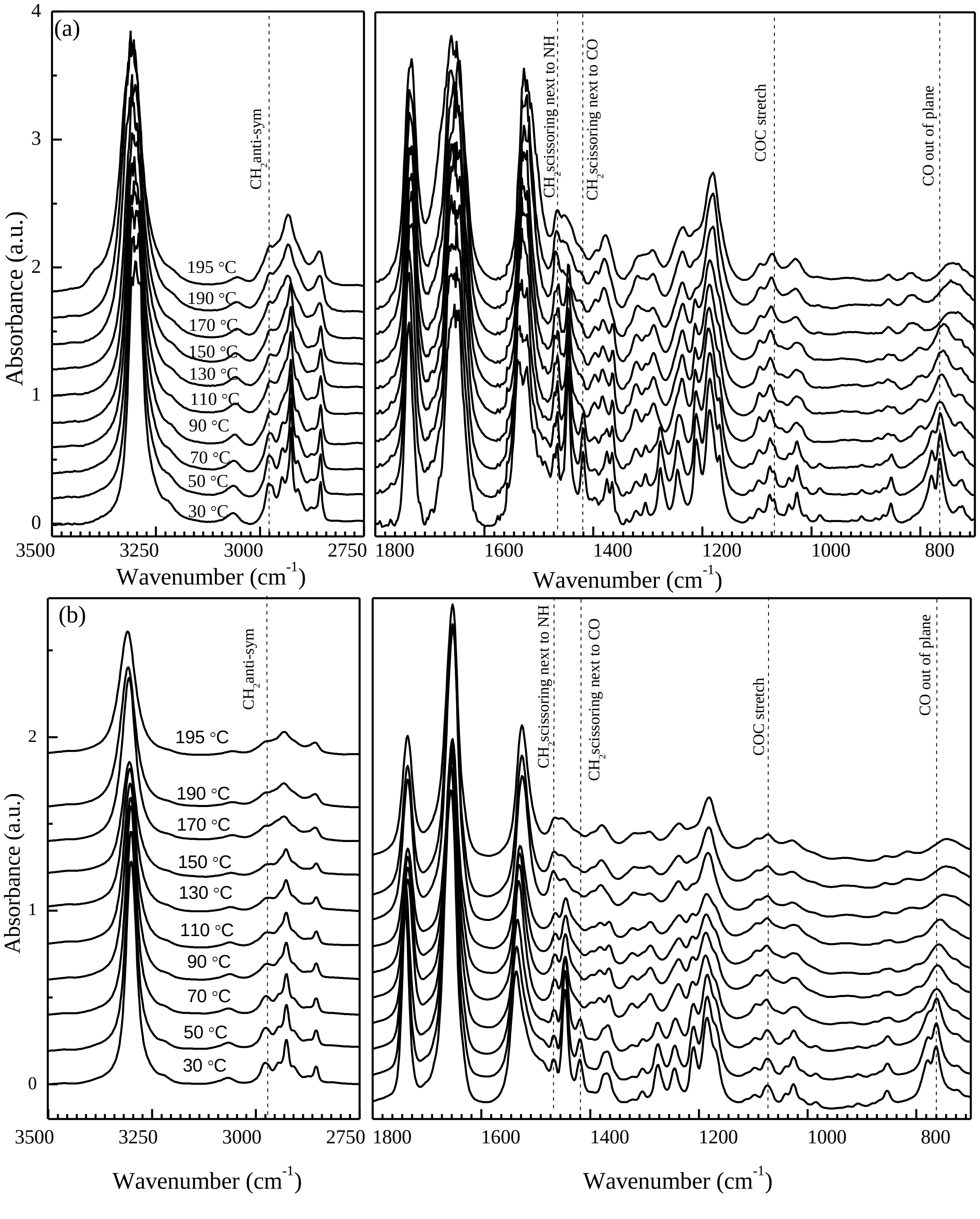

Figure S4. Variable temperature changes in the vibration modes of (a) melt and (b)water crystallized compound **1**.

With the onset of the transition T_{H2}, remarkable changes are observed in the methylene vibrational modes next to the NH group of the oxalamide groups. These changes arising due

to enhanced mobility of the methylene units between the oxalamide groups and are in agreement with the previously described solid-state NMR studies (see Figure 3).

Section S.4 Wide Angle X-ray Diffraction (WAXD).

WAXD was performed on water crystallized (WC) compound **1** and diffraction spectra were recorded in transmission mode, using a Bruker AXS HISTAR area detector installed on a P4 diffractometer, using graphite monochromatic CuKα radiation (λ = 0.154 nm) and a 0.5 mm collimator. The 2D data were subsequently background corrected and transformed into 1D profiles via integration.

Material studio software (*Accelrys Software Inc.*) was employed to calculate the crystallographic parameters. A Pawley refinement method was used to model the experimental WAXD data. The protocol used during modeling the WAXD pattern described below. The integrated patterns were subjected to Materials studio simulations program (*Accelrys Software Inc.*). After doing the background substation, indexing on the selected set of peaks is performed using a TREOR 90 program. In this trial and error based program primary solution for unit cell was searched from cubic to triclinic. The search ends as the Figure of Merit (FOM) criteria was fulfilled. The proposed unit cell was analyzed using a Pawley refinement iterative method for determination of lattice parameters. When the simulated data fits with the best possible values, a refinement is terminated and the refined lattice parameters were selected.

The crystal structure of water crystallized compound **1** has been studied using WAXD. The recorded diffraction pattern is used for determination of the unit cell and crystallographic planes. The TREOR 90 method in combination with a Pawley refinement is employed. The method adopted for calculation of the unit cell is described in the earlier work of and have been applied here [v]. Figure S5 depicts the comparison between the calculated and the simulated patterns. A good agreement between the calculated and the simulated patterns is

observed, which suggests that these crystals have a triclinic unit cell with dimensions *a* = 0.43 nm, b = 0.87 nm, c = 2.78 nm, α = 76.63 °, β = 85.15 ° and γ = 49.78 °. The calculated and simulated *d*-values with the corresponding miller indices are summarized in Table S4.

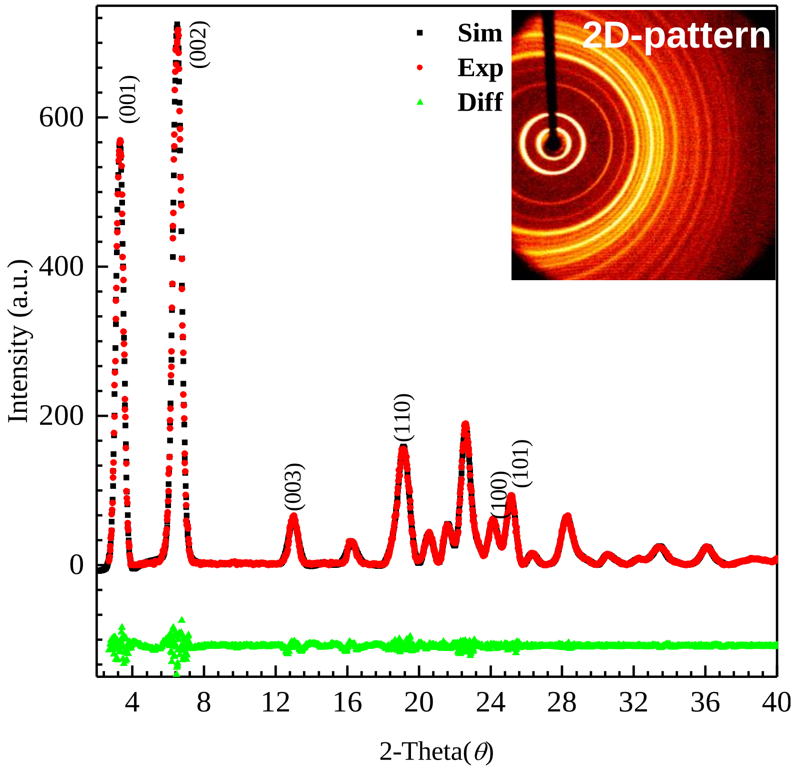

Figure S5. Experimental (black) and TREOR 90 simulated (red) WAXD pattern of water crystallized NA1. The green line in bottom shows the difference in the experimental and simulated pattern.

Table S4. Diffraction signals of water crystallized NA1, having triclinic unit cell at 30 °C.

Bravais-Miller indices			Water crystallized at 30°C		
h	*k*	*l*	d_{obs}	d_{sim}	Δ_{diff}
			nm	nm	nm
0	0	1	2.682	2.682	0.000
0	0	2	1.349	1.349	0.000
0	1	1	0.596	0.596	0.000
0	1	0	0.644	0.644	0.000
1	0	1	0.345	0.345	0.000
1	0	0	0.352	0.352	0.000
1	1	0	0.457	0.456	0.001
1	1	1	0.455	0.455	0.000

i Feike, M.; DEMCO, D.; Graf, R.; Gottwald, J.; Hafner, S.; Spiess, H. W. *J. Magn. Reson Ser. A* **1996**, *122*, 214–221

ii Saalwächter, K.; Lange, F.; Matyjaszewski, K.; Huang, C.-F.; Graf, R. *J. Magn. Reson.* **2011**, *212*, 204–215

iii Hayashi, S.; Hayamizu, K. *B., Chem Soc Jpn*, **1991**, *64*, 685–687

iv Morcombe, C.; Zilm, K. *J. Magn. Reson.,* **2003**, *162*, 479–486

v Harings,J. A. W., Yao, Y., Graf, R. van Asselen, O., Broos, R. & Rastogi S. *Crystal Growth & Design*, **2008**, *8*, 2469-2477
